# The “Bald Disease” of the Sea Urchin *Paracentrotus lividus*: Pathogenicity, Molecular Identification of the Causative Agent and Therapeutic Approach

**DOI:** 10.3390/microorganisms11030763

**Published:** 2023-03-16

**Authors:** Serena Federico, Francesca Glaviano, Roberta Esposito, Enea Tentoni, Pasquale Santoro, Davide Caramiello, Maria Costantini, Valerio Zupo

**Affiliations:** 1 Stazione Zoologica Anton Dohrn, Department of Ecosustainable Marine Biotechnology, Villa Comunale, 80121 Napoli, Italy; 2 Stazione Zoologica Anton Dohrn, Department of Ecosustainable Marine Biotechnology, Ischia Marine Centre, 80077 Ischia, Italy; 3 Department of Biology, University of Naples Federico II, Complesso Universitario di Monte Sant’Angelo, Via Cinthia 21, 80126 Napoli, Italy; 4 Centro Ricerca Sperimentale APA-CT S.r.l., Via N. Sacco 22, 47122 Forlí, Italy; 5 Diagnostica di Laboratorio (Di.Lab.) S.r.l., Via Beccadelli, 25, 80125 Napoli, Italy; 6 Stazione Zoologica Anton Dohrn, Department of Marine Animals Conservation and Public Engagement, Villa Comunale, 80121 Napoli, Italy

**Keywords:** bald disease, bacteria, epidemics, echinoid necrosis, sea urchin

## Abstract

In recent decades, various species of Mediterranean sea urchins, including *Paracentrotus lividus*, have been subject to widespread seasonal episodes of mass mortality whose causative agents are still unclear. In particular, *P. lividus* is subject to late winter events of mortality, due to a disease manifested by a massive loss of spines and the presence of greenish amorphous material on the tests (i.e., the sea urchin skeleton consisting of spongeous calcite). Documented mortality events show a seasonal epidemic diffusion and might produce economic losses also in aquaculture facilities, besides the environmental constraints to its diffusion. We collected individuals showing conspicuous lesions on the body surface and reared them in recirculated aquaria. Samples of external mucous were collected along with coelomic liquids and cultured to isolate bacterial and fungal strains, further submitted to molecular identification through the amplification of prokaryotic 16S rDNA. In addition, pools of infected sea urchins were reared in recirculated tanks after short baths in a formulated therapeutic compound and their survival rates were compared to non-treated individuals for variable periods. Here, we aimed at a redescription of the etiopathogenetic nature of the parasites and tested the efficacy of a possible treatment, to be proposed for aquaculture purposes.

## 1. Introduction

Several opportunistic microorganisms occur naturally over the body or in the gut of echinoderms. This is the case of gut-associated bacteria of some regular echinoids [1] and of subcuticular bacteria found in other echinoderms [2]; they are normally non-pathogenic. Inversely, the coelomic fluids of echinoderms are normally sterile in healthy specimens, due to the antimicrobic activity of specialized phagocytic coelomocytes [3]. The activity of coelomocytes is not limited to phagocytosis, since some coelomocytes in various species of echinoids may release mucins, compounds that promptly immobilize microorganisms entering the coelom [4]. Other echinoids are provided with special *spherulae* cells that produce such antibacterial substances [5,6,7] as naphthoquinone, a pigment belonging to the group spinochrome (echinochrome) [7]. However, some microorganisms are able to invade the coelomic liquids in specific cases, due to seasonal variations of the echinoderm’s physiology or to physical damage to their bodies. For example, in *Asterias forbesi* the coelomic fluids may be invaded by various opportunistic bacteria when it undergoes autotomy or it is physically injured. In other cases, bacteria may be present in the coelomic fluids due to well-documented diseases, naturally impacting the populations of sea urchins, as in the case of so-called “bald disease”. In fact, Jangoux [8] documented various regular echinoids suffering from this impressive disease, whose main symptoms are represented by evident lesions of the body surface, followed by the appearance of green spots on the test. Other symptoms are represented by extensive losses of spines, the necrosis of dermal tissues and total loss of the epidermis, up to totally denuded tests [9,10]. Often, lesions may ultimately lead to test perforation. In this case, large death events occur [11]. In other cases, the lesions may be spatially limited (less than 30% of the body surface) and in this case the sea urchins may spontaneously recover [9]. Bald disease has been recorded by various authors in several areas of the world, such as California (on *Allocentrotus fragilis* by Giese [12] and on *Strongylocentrotus franciscanus* and *S. purpuratus* by Pearse and Hines [13]), along the French coasts of the Mediterranean (on *Arbacia lixula* by Höbaus et al. [14] and on *Cidaris cidaris* by Maes and Jangoux [9]), off the N.E. Atlantic Brittany coasts (on *Echinus esculentus* by Nichols [15]), along all the coasts of the Mediterranean (on *P. lividus* by Maes et al. [10]), in the English channel (on *Psammechinus miliaris* by Maes et al. [10]), in the western Mediterranean (on *Sphaerechinus granularis* by Höbaus et al. [14]), in Nova Scotia (on *Strongylocentrotus droebachiensis* by Scheibling and Stephenson [16]). In addition, damage described as “spotting disease” has been reported in various breeding and culturing locations in Japan by Shimizu et al. [17], with similar lesions and external symptoms. In this case, diseased sea urchins had a unique lesion on their bodies, characterized by a blackish-red color, the separation of spines and failure in the attachment of tube feet. These diseases were generically named “spotting disease” and were characterized by spots of variable colors (reddish to blue and green) and the loss of spines. Consequently, all species of echinoids, at any latitude, appear capable of contracting these diseases, although the specific factors facilitating their diffusion are still unknown.

Bald disease (as well as spotting disease, which might correspond to the same etiological factor) produces a well-known series of events [8] and induces a conspicuous inflammatory-like syndrome around the infected spots, involving a massive migration of coelomocytes (phagocytic cells and red spherule cells) around and within the infected areas [10,18]. Interestingly it is not species-specific and, in fact, it has been displayed by several species of sea urchins at a range of latitudes. Furthermore, this disease may be experimentally transferred from one species to another [9]. The similarity of symptoms induced previous authors to hypothesize that an identical micro-organism could induce such generic signs, although there is still confusion even on the actual etiological factor. Shimzu et al. (2020) [17] indicated the protozoan, *Paramoeba invadens*, as the causative agent of the described histopathological lesions. In addition, Hernàndez et al. [19], in their studies on the mass mortality that affected eastern Atlantic populations of the barren-forming sea urchin *Diadema africanum*, isolated the amoeba *Paramoeba brachiphila* as the only pathogen from the moribund and dead sea urchins, suggesting that this amoeba might be the primary cause of mortality. Furthermore, other studies indicated various bacteria as the possible causative agents. At the moment, there are uncertainties on the role of microorganisms found on test lesions because they could represent secondary effectors of a primary parasite, still unidentified. All this makes this matter quite puzzling and still debated.

Special attention should be dedicated to the epidemic transmission. These diseases are easily communicable when pieces of necrotic tissue are painted on the body of healthy individuals over experimentally produced injuries [11]. Previous authors also detected 14 different bacterial strains on the lesions found in *S. purpuratus*. However, only two strains out of fourteen were able to initiate lesions when experimentally injected in sea urchins, i.e., *Vibrio anguillarum* and *Aeromonas salmonicida*. It was also demonstrated that a specific stress, such as a mechanical injury, was needed to initiate the transmission and produce the characteristic lesions, but this point is still debated. In fact, this theory (the accidental input of bacteria due to injuries) may not explain the seasonal rhythms of the disease in nature and in some specific environments. Evidently, bald disease may kill up to 75% of *Paracentrotus lividus* in some seasons and years [20] and its incidence is higher in shallow waters and in summer [21]. Girard et al. [22] reported how high sea surface temperatures and low wave heights can be conditions that trigger outbreaks of disease in the sea urchin *P. lividus*. Epizootics of *Strongylocentrotus droebachiensis* in the NW Atlantic and NE Pacific, and *P. lividus* and *Diadema antillarum* in the eastern Atlantic, have been linked to climate changes and the overfishing of sea urchin predators, as reported by Feehan and Scheibling [23]. Very recently, Salazar-Forero et al. [24] confirmed the relationship between winter storms and sea urchin marine pathogen dynamics. In fact, they monitored the marine pathogen assemblage before and after a winter storm on Tenerife Island on different habitats including sea water, sediment and algae, and in four species of sea urchin hosts, *D. africanum*, *Arbacia lixula*, *P. lividus* and *Sphaerechinus granularis*. Six pathogens were identified, including *Neoparamoeba branchiphila* (in the coelom of all four sea urchins studied), *Vexillifera minutissima*, *Acanthamoeba* sp., *Vahlkampfia* sp., *Vibrio alginolyticus* (occasionally detected in three sea urchin specimens) and green colonies of *Vibrio* spp. Furthermore, *V. alginolyticus* was involved in the disease provoking a widespread mass mortality event of the sea urchin *D. africanum* in the subtropical eastern Atlantic [25].

In other species and in other areas of the world the epidemics may exhibit dissimilar patterns. Consequently, the causative agent is still debated, in spite of the remarkable consistency in the presence of the two species reported above. For example, Miller and Colodey [26] showed mass mortalities of *Strongylocentrotus droebachiensis* with typical symptoms of bald-disease along the Atlantic coasts of Canada. They were not due to bacterial agents, because bacteria could be a secondary factor associated with mass mortalities [11], initiated by a still unidentified (maybe mycetes or cyanobacteria) pathogen that caused the lysis of tube feet and loss of spines. In this regard, it is known that the blue-green alga *Dactylococcopsis echini* is able to produce, on *Echinus acutus*, specific patches on the denuded test, showing a typical blue-green color that might resemble the color of the green patches found in Mediterranean urchins infected by bald disease [27]. The cyanobacterium was isolated from smears of necrotic tissues, but it was not recognized in histological sections. Although the pathogenicity of *D*. *echini* remains to be confirmed, its symptomatology is similar to that described for bald disease. In fact, it destroys the calcified tissues and produces an intense reaction of amoeboid cells, which massively invade the necrotic areas, as mentioned for bald disease. However, the typical external signs of both spotting disease and bald disease are the focal lesions on the test surface and the separation of spines, followed by a progressive disfunction of spines and *pedicellarie* movements, probably caused by the destruction of muscles. In all these pathological displays, eleocytes are concentrated in the dermis–epidermis around the spotted areas (generally blue-green or reddish) and the discolored areas. The authors debated if the causative agent was an amoeba (*P*. *invadens*, as in spotted disease) or some bacteria (*Vibrio* sp. and *Aeromonas* sp., as in bald disease).

Here, we investigated several individuals of *P*. *lividus* collected in the Bay of Naples (Italy) with the hypothesis that the primary causative agent could be a cyanobacterium (e.g., *Dactylococcopsis echini*, as observed in *Echinus acutus*), common to both diseases, and that the microorganisms further sustaining the infection could be secondary infective agents, seasonally or locally present and predominantly affecting selected species of sea urchins. In fact, hypotheses on the dependence of the parasite on higher temperatures seem unconvincing, given the continuous global increase in temperatures that scarcely fits with the intermittent appearance of parasitized echinoids in various areas of the world. In contrast, cyanobacteria are known to locally produce periodic blooms and their intermittent increases in various areas could be realistic. Finally, we attempted a therapeutic treatment to facilitate the recovery of sea urchins in captivity, to prevent the economic losses in production plants in case of epidemic events spreading in culture tanks.

## 2. Materials and Methods

This research was conducted in the frame of an investigation aimed at producing effective culture techniques for *Paracentrotus lividus*. Since the periodic infections of tests are relatively frequent [21], and they have become even more frequent in the last decade, probably due to global changes [28], the sea urchins collected monthly for 12 months were accurately monitored to detect any symptoms of disease and the seasonal patterns of abundance of parasitized sea urchins within the natural populations living in the Bay of Naples. The diseased sea urchins collected were submitted to the analyses described below in order to define: i) the etiology and the organs mainly influenced by the parasite; ii) the parasites responsible for the symptoms; iii) the means for the spread of the infections; iv) a possible therapeutic treatment to be applied in aquaculture productions, in case of accidental spread of the infections. The experimental methods needed to answer each of the above questions are reported here. Plate cultures of possible parasitic agents and the molecular identification of strains were applied because previous investigations [9], mainly based on histological approaches, were unable to clarify the actual responsible agent for bald disease due to the concomitant presence of various opportunistic microorganisms making the determination quite complex. In contrast, here we tried to isolate the strains of microorganisms from body lesions and test their ability to trigger the appearance of the symptoms.

### 2.1. Ethics Statement

*P. lividus* (Lamarck) were collected from a location in the Bay of Naples that is not privately owned or protected in any way, according to the Italian Laws (DPR 1639/68, 19 September 1980 confirmed on 10 January 2000). Field collections did not include endangered or protected species. All experimental procedures on animals were in compliance with the guidelines of the European Union (Directive 609/86) and the rules of Stazione Zoologica Anton Dohrn on animal welfare.

### 2.2. Collection of Sea Urchins

Specimens of *P. lividus* were collected in the Bay of Naples, over transects ranging from the eastern sector of the harbor of Napoli (Rocce Verdi: 40°47′51″ N, 14°11′55″ E) to the Island of Procida (Point Schiavone: 40°45′52″ N, 14°02′13″ E) at depths ranging between 5 and 15 m, on rocky shores and seagrass meadows. Specimens were manually collected by scuba divers in order to obtain approximately 200 individuals per month, when possible, from October 2019 to September 2020. Specimens were collected in order to provide healthy individuals for in vitro fertilizations and they were individually inspected under a stereomicroscope to prevent the introduction of parasites in the culture tanks. The size of collected specimens, in all samples, ranged from 35 to 54 mm (maximum diameter measured by a caliper). Since the specimens were manually collected to be used for in vitro fertilization, only individuals of reproductive size were selected by scuba divers, while smaller individuals were immediately discarded. For the same reason, the monthly collections performed cannot be considered quantitative, not being related to a given surface area. The evaluation of healthy and diseased individuals was consequently computed as a percentage (diseased over healthy) to indicate the monthly presence and frequency of diseased specimens in the field, as referred to the reproductive size of the stocks. In particular, specimens showing such disease symptoms as loss of spines and colored or whitish spots on their tests were periodically detected during the inspection and they were promptly selected and isolated in recirculated rearing tanks, to be submitted to further experimentation.

### 2.3. External Analysis and Microorganism Cultivation

All diseased specimens, immediately after the detection, were measured, photographed and analyzed under a stereomicroscope to reveal early symptoms of disease. Fifteen individuals (five in each month of their presence, with the exclusion of June, when they were insufficient for such analyses) were dissected to evaluate damage to the gut, gonads and other internal parts. Samples for bacterial isolation were collected both from the body surface (by scraping the colored spots) and the coelomic fluids of all diseased sea urchins, as well as from similar areas in 15 apparently healthy sea urchins (not showing any disease symptom) from the same collections. In the correspondence of the colored spots the epidermis was damaged and covered by a mucus/gelatinous layer that was collected by a scalpel. The following media were used for bacterial isolation and to produce colonies to be submitted to further analyses: Marine Agar (Dehydrated Culture Media, Applichem) for microbiology, a medium for the cultivation, isolation and maintenance of a wide variety of heterotrophic marine bacteria, with the following composition (g/L): Boric Acid: 0.022, Ammonium Nitrate: 0.0016, Calcium Chloride: 1.8, Strontium Chloride: 0.034, Yeast Extract: 1.0, Iron Citrate: 0.1, Magnesium Chloride: 8.8, Peptone: 5.0, Potassium Bromide: 0.08, Potassium Chloride: 0.55, Sodium Chloride: 19.4, Sodium Fluoride: 0.0024, Sodium Hydrogen Carbonate: 0.16, di-Sodium Hydrogen Phosphate: 0.008, Sodium Silicate: 0.004, Sodium Sulphate: 3.24, Agar: 15.0, pH: 7.6 ± 0.2. In addition, we used pre-confectioned Sabouraud CAF Agar (plates, cat. n° 11035), Mannitol Salt Agar (plates, cat. n° 10030), MacConkey agar (plates, cat. n° 10029), Chromatic detection agar (plates, cat. n° 11611), Columbia CNA agar (plates, cat. n° 11024) obtained from Liofilchem S.r.l. (TE. Italy). All culture plates were incubated one week in a thermostatic chamber with a 12:12 photoperiod at 18 °C prior to starting the analyses of strains.

### 2.4. Molecular Identification of Strains

The amplification of prokaryotic 16S rDNA was performed by using the universal bacterial primers 27F-1385R [29,30], which amplify approximately 1400 bp fragments of bacterial 16S rRNA gene sequences. Amplification was performed on bacterial colonies collected on the culture plates, using the Lyses and PCR-Go kit (DNATech, Spin-off, Naples, Italy), by picking one colony superficially from the agar plate and following further manufacturer instructions. The amplified fragments were purified from agarose gel using QIAquick Gel extraction kit (Qiagen, Hilden, Germany) and the specificity of PCR products was checked by DNA sequencing. Sequences were compared to those present in databases, using the Basic Local Alignment Search Tool (BLAST) algorithm (available at www.ncbi.nih.nlm.gov) to identify known sequences with a high degree of similarity.

### 2.5. Experimental Infection

To investigate the method of transmission of the disease, two replicates of five diseased sea urchins collected in February were kept in 45 L tanks along with two healthy sea urchins collected from rearing tanks and deriving from previous collections, after at least 30 days of accurate monitoring, to confirm the absence of external symptoms. The diseased sea urchins were pooled in the same tanks with healthy sea urchins for 15 days and they were easily recognizable, based on the spatial distribution of spots present on their tests. All specimens were inspected daily to monitor the appearance of disease signs and their progression over time. Five other diseased sea urchins were kept in two individual tanks that were connected, through a Recirculating Aquaculture System (RAS), to other tanks hosting healthy sea urchins. These specimens were inspected daily to monitor the appearance of disease signs.

### 2.6. Experimental Treatment

Three replicates of 5 diseased sea urchins (at various stages of the disease, from the presence of a few small whitish spots and the loss of spines covering less than 20% of the test, up to the most heavily infected individuals with more than 80% of the test covered with colored spots and most spines lost) were kept in glass tanks (45 L volume, equipped with an external canister filter Sicce Whale, in a thermostatic chamber at 18 °C) along with three replicates of healthy sea urchins, kept in the same conditions, in parallel. After 24 h, the diseased sea urchins were collectively transferred to a therapeutic bath, contained in a 3 L glass beaker. The bath was prepared by pouring 50 µL/L of Greenvet Gill-Fish Professional (APA-CT srl, Forlì, Italy) in filtered seawater. Gill-Fish Professional is a liquid mixture containing compounds obtained from plant processing (*Agrimonia eupatoria, Plantago major, Calendula officinalis, Citrus lemon, Echinacea angustifolia, Eucalyptus globosus, Glycyrriza glabra, Melaleuca alternifolia, Origanum vulgare, Solidago virgaurea*). The components of this mixture exhibited antibiotic and antifungal properties against microorganisms frequently producing diseases in cultured fish and invertebrates. The “Gill Fish” blend is industrially produced and suitable for supporting animal welfare in aquaculture and pet technology. To test the effects of the plant blend, sea urchins were taken into the bath beaker for 15 min and then transferred back to their rearing tank. The treatment was repeated after 3 days. In the next 60 days, all experimental tanks were daily inspected to check the presence of disease symptoms and the eventual regression of external signs of disease, as well as the ability of reared specimens to feed on small leaves of *Posidonia oceanica* offered ad libitum. Mortality rates and recovery rates were recorded after 10 days, 20 days and 60 days. Treated sea urchins, at the end of the recovery period, were re-stoked in culture tanks and further submitted to in vitro fertilization.

## 3. Results

### 3.1. Occurrence of Diseased Sea Urchins

**Figure 1:**
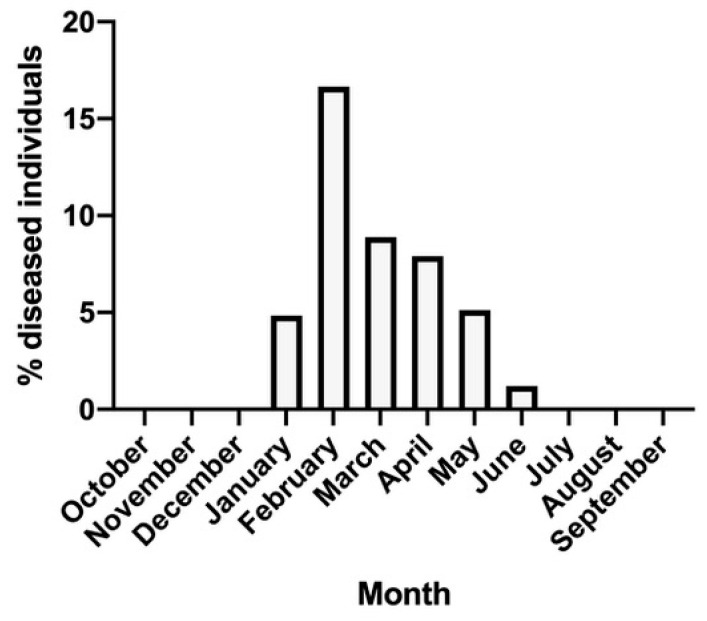
Percent of diseased individuals found in the samples monthly collected in the bay of Naples. In August no collections were performed, for technical reasons.

Diseased sea urchins were retrieved in various samples (Table S1) and in variable abundances and they seasonally accounted for 3.2–15.6% of each sample randomly collected by scuba divers. Most diseased individuals were found in late winter collections (February–March; Figure 1), with a peak of 32 diseased individuals detected in February, over a collection of 200 specimens. In contrast, early winter and summer samples, from July to December, did not contain diseased individuals. Individuals collected from April to June exhibited weaker symptoms (whitish spots, a few missing spines, the absence of greenish spots) and frequently recovered spontaneously, after isolation in rearing tanks with recirculated water. Infected individuals collected at the end of the winter, in February–March, exhibited more severe symptoms, an immediate increase in the diseased areas and rapidly deceased after they were reared for 7–10 days in recirculated tanks kept at 18 °C in a thermostatic chamber. Almost all healthy individuals collected in all months exhibited good physiological condition even 12 months after the collection, being reared in RAS tanks in perfect conditions for reproductive purposes. However, besides the individuals collected with evident disease symptoms (described above), seven individuals recorded as “healthy” after previous collections and reared in RAS tanks for 8 months in a thermostatic chamber at 18 °C with a 12/12 h photoperiod exhibited initial symptoms of disease. They were detected in the course of daily inspections routinely performed and promptly isolated in May. In this month, diseased individuals were not abundant in the field (about 5.7%), although still present (Figure 1).

**Figure 2:**
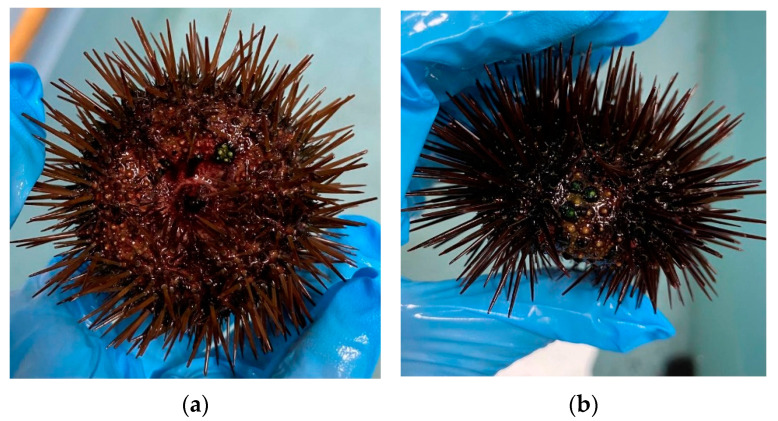

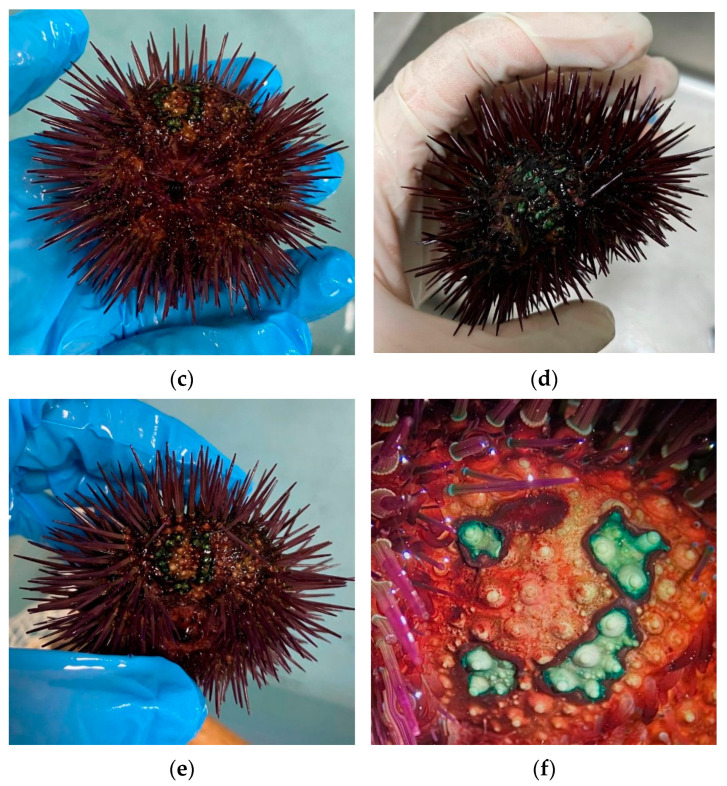
Specimens bearing external symptoms of bald disease at various stages. (**a**) Initial appearance of the disease is indicated by a slight loss of spines in the aboral region. (**b**) Lateral side contains a first appearance of whitish spots and loss of spines. (**c**) Extensive loss of spines and enlargement of diseased areas on the test. (**d**) Appearance of blue spots and initial corrosion of the test on the lateral side. (**e**) Extensive loss of spines and widening of the green spots. (**f**) Appearance of green spots and of a corroded area (dark spot) on the test observed under the stereomicroscope at 30×.

The isolated sea urchins exhibited a range of symptoms (Figure 2), from the loss of a few spines in areas of the tests smaller than 5% of the total surface (Figure 2a,b), showing also whitish spots and slower movements (Figure 2c), up to heavy and diffused damage covering more than 80% of the test surface (Figure 2d,e), with various greenish or blue-green rounded spots in some areas (Figure 2f). Sometimes, the test appeared perforated and, in this case, the internal tissues appeared damaged and seriously infected. In the worst cases, the sea urchins were still alive (slight movements of the pedicels) but unable to stably attach to the glass surface.

### 3.2. Identification of Bacterial Strains

Eight different bacterial colonies were isolated from the body surface of diseased sea urchins, for which a blast similarity search revealed that six showed the highest percentage of identity with *Tenacibaculum* species (Table 1). The analysis of the coelomic fluids of diseased sea urchins revealed the presence of three bacterial colonies, two of which were also ascribed to *Tenacibaculum* sp. In the control specimens, bacterial colonies isolated both from the body surface and the coelomic fluids were represented by four genera. Only one colony ascribed to *Tenacibaculum* sp. was isolated, externally, from the body of control sea urchins, in the absence of evident disease symptoms. This bacterial strain was never found in the coelomic fluids of the control sea urchins. Some species of bacteria, such as *Pseudoalteromonas*, *Tenacibaculum* sp., *Vibrio* sp. and *Vibrio cortegadensis,* were specifically present in healthy specimens, on the external surface of tests and in their coelomic fluids. In contrast, *Tenacibaculum* sp. strains and one strain belonging to *Vibrio* were largely isolated on diseased specimens, both on the external surface of sea urchins and in their coelomic fluids.

### 3.3. Experimental Infection

Specimens experimentally injected with coelomic fluids and small portions of diseased tissues scraped from the surface of tests of diseased sea urchins never showed disease symptoms. Specimens injected with bacterial colonies grown and collected from Petri dishes (further re-suspended in sterile seawater) never showed disease symptoms. Healthy specimens reared in tanks connected to the same circulation system in common with tanks containing diseased specimens never showed the appearance of disease symptoms.

However, individuals reared in the same tank with diseased specimens (engaging in physical contact) consistently started showing the typical symptoms of bald disease (white spots, loss of spines, greenish spots) in periods ranging from 5 to 9 days from the beginning of the test.

### 3.4. Treatment and Recovery

**Figure 3:**
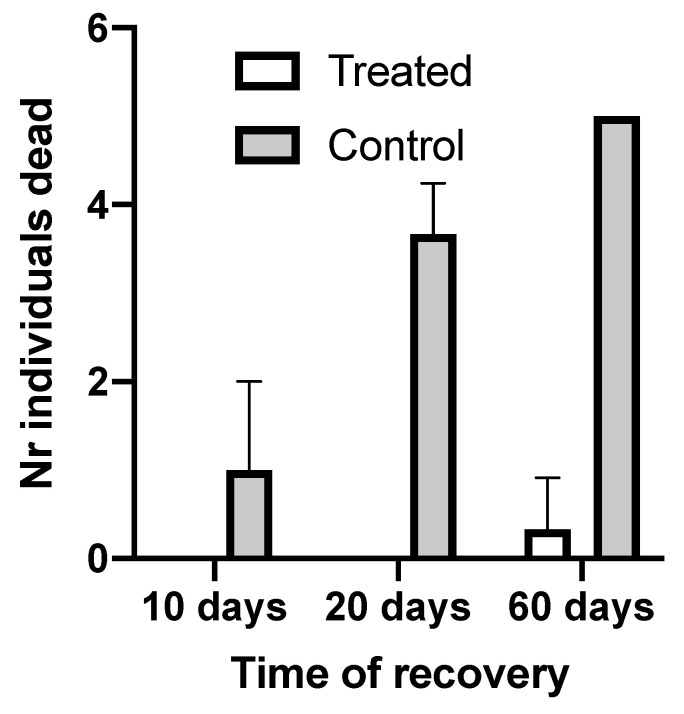
Mortality rates recorded in treated (single bath of 15 min) and control specimens, as averages of three replicates of five individuals each. The readings were performed three times during the recovery period, with clean seawater in recirculated tanks. Averages were evaluated on three replicates of five individuals, along with standard deviations among replicates (indicated by vertical bars).

Infected individuals were divided into groups of five specimens, reared in three replicated tanks and submitted to a 15 min treatment with the plant-derived therapeutic Gill-Fish as indicated in Methods. The monitoring of their health status indicated that immediately after the treatment all specimens appeared lethargic. They were all restored to their tanks with the oral side up, and some of them remained for several minutes on a lateral side, unable to complete the turning of their bodies to the correct position. However, their pedicels were actively moving and they appeared alive. All treated individuals in all replicates, besides a single case, showed complete recovery after 10 days post-treatment (Figure 3 and Table S2). In a single tank, one individual previously showing complete recovery again exhibited symptoms of disease and died after about one month. Mortality rates in control tanks (diseased, non-treated) were low after 10 days (only 20%) but all non-treated individuals showed more serious symptoms of disease, with the significant presence of green spots and lost spines left on the bottom of tanks. After 20 days, most non-treated individuals died in the control tanks and after 60 days 100% mortality was recorded, as compared to the almost total recovery of treated individuals (Figure 3). No individuals in control tanks restarted feeding on maize and *P. oceanica* leaves administered daily. Moreover, treated individuals did not feed during the first 10 days post-treatment. After this period, they started to consume the maize and the leaves daily offered ad libitum. Other additional experiments, which are not reported in this study, were performed. In particular, one week after the first experiment, the individuals that still did not show feeding activity were treated once again to obtain a faster recovery, and none of these treated individuals died. In addition, we attempted a treatment (not reported in this study) with a double dose of the therapeutant (100 µL/L seawater for 15 min) and it did not produce any mortality, demonstrating that the effective dose could even be doubled. However, treated individuals showed contracted pedicels for a few days after the double-dose treatment and did not feed for several days. After 7 days, they all recovered and were finally transferred to culture tanks.

## 4. Discussion

The wide and consistent presence of diseased sea urchins seasonally found in samples collected in the Bay of Naples confirmed the diffusion of bald disease in the Mediterranean and demonstrated the need for the identification of the actual causative agent. Our data also confirmed the seasonality characterizing this disease [31] and the similitude of the symptoms prompted by bald disease and spot disease, indicating that the two sets of external signs might be ascribed to the same causative agent [31]. Given the seasonal increase in February–March, when field temperature was still low, and also the presence of diseased individuals in culture tanks kept at a constant temperature of 18 °C, it appears likely that a direct effect of temperature on the progression of the epidemic disease may be excluded [9]. However, our data indicated that the diseased individuals were consistently concentrated in some early spring months, according to a bell-shaped trend that would indicate the seasonality of their appearance as an important feature. The influence of food webs [32] and of mass concentrations of specimens in reproductive areas [33] cannot be excluded. Mass mortalities were also observed with seasonal rhythms in eastern aquaculture plants [17], demonstrating that several seasonally occurring factors may influence the onset and the expansion of these epidemic diseases. Previous findings could indicate a multi-factorial origin of the disease, influenced by a peculiar coincidence of physiological conditions (sea urchins are at the end of their reproductive season), environmental conditions (seasonal changes of temperatures, storms, planktonic microorganism blooms) and food web cycles (the re-start of algal growth, increase in daily irradiation; [31]). However, it is also known that some microorganisms, such as cyanobacteria, are subject to seasonal cycles of abundance [34]. For example, Almanza et al. [35] observed that the trophic state of water bodies, according to seasonal cyclical rhythms, deeply influences the frequency and the distribution of cyanobacterial blooms. Complex patterns of environmental factors, such as temperature and the concentration of nutrients, rule their expansion and regression. The presence of cyanobacteria, in turn, impacts the ecology of several aquatic systems, although eutrophication and global warming, recorded in recent decades, have triggered an increase in bloom frequency in various areas of the world. Nevertheless, the presence of parasites inducing “bald disease-like” effects has been documented since the last century and [20] an abrupt decline of the sea urchin population in Port-Cros due to such epidemics has been recognized. These observations would be in agreement with the hypothesis that a cyanobacterium, such as the blue-green alga *D. echini*, might represent the initial trigger for the start of the infection, as initially proposed by Mortensen and Kolderup [27].

Here, we confirmed that bald disease is transmitted by the direct contact of infected individuals with other sea urchins and that simple connection by RAS circulating water is insufficient to trigger the onset of the disease. Our observations are in line with those of previous studies by Maes and Jangoux [9], which reported a similar succession of events and tissue damage. We also confirmed that, once started, the disease proceeds up to the death of infected individuals, because the last stages of the disease, involving the corrosion of the tests and infection by several species of bacteria, have lethal effects [16]. However, a few individuals collected in early spring showed mild symptoms of the disease, with smaller patches and the absence of greenish spots and they were occasionally left in a control tank, spontaneously recovering after about 30 days in the absence of specific treatments (personal observation). This observation confirms the seasonal occurrence of the disease, indicating that not only the initial events, but also further phases of the disease (including recovery), may be influenced by seasonal rhythms, even if the etiology responsible has not yet been fully assessed [9]. This finding is in line with the patterns of diseased individuals found in our collections, showing a clear maximum in February and a bell-shaped distribution reaching from late winter up to late spring. As indicated above, various hypotheses have been proposed, indicating protozoans [36] or bacteria [9,11,37,38] as the possible causative agents, but also cyanobacteria have been proposed as a possible etiology responsible [27,39].

The symptoms observed [10,18] in the specimens collected in the Bay of Naples (Italy) coincide with those described by previous authors [8,21,40]. In particular, we observed, in sequence, according to Jangoux [8]: (i) the epidermis at the base of some spines turned greenish; (ii) spines were lost and the green epidermis along with its underlying dermal tissue became necrotic; (iii) the epidermis and superficial dermal tissues were lost and circular denuded areas were formed on the test; (iv) the upper layer of the test was progressively corroded and in some cases through-holes were observed. It is worth observing that such a sequence of symptoms was observed both in bald disease [18] and spotted disease [17]. This finding would endorse a hypothesis of coincidence of the two diseases, described by various authors in sea urchins from several areas of the world [8]. However, the assemblages of microorganisms found in various environments are quite diverse, although several genera are likely to be consistently present. For example, previous authors detected other possible pathogens, such as *Vibrio splendidus* [38] and *Vibrio alginolyticus* [25], which were absent in the strains we collected, but the genus *Vibrio* was consistently present in our samples. Such variability creates uncertainty about the nature of the actual causative agent and the role of the microorganism associations that take part in the infection process. In addition, previous authors [11] demonstrated that only the isolates of *Vibrio anguillarum* and *Aeromonas salmonicida* (two well-known pathogenic marine bacteria) were sufficient to initiate the formation of lesions in the laboratory. In our case, the strains isolated from the tests differed from those isolated in the coelomic fluids, but *Tenacibaculum* spp. dominated the bacterial associations found in diseased sea urchins, while only a single colony was found in healthy sea urchins, and only externally, in agreement with previous studies on various echinoderms [2]. Furthermore, one strain of *Vibrio* sp. was specifically and largely isolated from diseased specimens, although other strains of *Vibrio* spp. were also present in healthy sea urchins. Some of these strains were present in the coelomic fluids of healthy specimens. These fluids should be supposed to be free of microorganisms, due to the presence of natural defenses and coelomocites [4,8]. Consequently, we cannot exclude that specimens hosting *Vibrio* spp. in the coelom were already infected but not yet showing external symptoms.

*Vibrio* is a genus of ubiquitous bacteria found in a variety of marine habitats and contains about a hundred described species (twelve of which cause infections also in humans) and these Gram-negative, rod-shaped bacteria are natural constituents of all aquatic systems, often being degraders or opportunistic parasites [41]. Some *Vibrio* spp. found in marine ecosystems induce wound infections [42]. Remarkably, also in our case two bacteria, namely *Tenacibaculum* spp. and *Vibrio* sp., were consistently present in diseased sea urchins and totally or almost absent (according to the species) in healthy sea urchins. In addition, ocean warming, characterizing the ecology of seas in the last decades, was demonstrated to increase the spread of pathogenic *Vibrio* spp. [43] and this is in line with the spread of sea urchin diseases, reported in the literature and confirmed in this study. Moreover, vibriosis may be seasonally associated with heat waves [28,38] and this trend is also in agreement with the seasonal patterns of occurrence of the diseased urchins in the Mediterranean.

This cannot be considered as the proof they are the actual etiological agents, because other microorganisms could prime the process and environmental pressures could contribute to its activation [19,24]. In fact, when we injected coelomic fluids collected from the bodies of diseased individuals into the bodies of healthy sea urchins, these were unable to prompt an infection. In contrast, the direct contact of healthy and diseased sea urchins was sufficient to initiate the process in the course of a few days. This evidence, and the demonstration that the parasite is not transmitted through a RAS system, indicates that a direct contact is necessary for the spread of the epidemic, as also suggested by other research. For example, Jangoux [8] concluded from experimental infectivity tests that a stress such as a physical injury is necessary for the formation of characteristic lesions. They demonstrated that a small fragment of infected test, glued on experimentally injured tests, was sufficient to prompt the symptoms of bald disease. Conversely, as indicated above, previous studies [42] indicated the possible role of a cyanobacterium, although it has not been identified in more recent research.

We also demonstrated that a plant-derived therapeutic was able to fight the disease and led diseased sea urchins to a total recovery, if the damage to their tests was not too serious (spots covering less than 30–40% of the test surface). This result will be important in developing possible treatments for cultured sea urchins and other echinoderms of commercial interest, given the wide infectivity demonstrated by these diseases, impacting the natural populations of such different echinoderms as Asteroidea, Holothuroidea, sea urchins and Crinoidea [7,44,45]. The therapeutic was demonstrated to be highly effective, producing 100% recovery if the sea urchins were not too heavily infected, and the recovery was complete, because the recovered individuals were further successfully submitted to in vitro fertilization. This represents a very important finding, given the growing importance of echinoculture activities and the economic losses potentially due to such epidemic diseases.

## 5. Conclusions

In conclusion, this investigation indicated that the bald disease and spotted disease of sea urchins are likely to correspond to the same pathology and they are characterized by a similar succession of heavy symptoms. We also demonstrated that some species of bacteria, indicated by previous studies, such as *Vibrio anguillarum* and *Aeromonas salmonicida*, along with other opportunistic bacteria, such as *Tenacibaculum* spp. identified during this experimental study, might be the main invaders when the epidemic is in a mature stage of development. However, the microorganism able to prime the process, probably facilitated by peculiar environmental conditions, could be different and it is feasible that a still unidentified cyanobacterium might be the actual etiological factor triggering the disease, although direct contact among diseased and healthy individuals is indispensable. Unfortunately, we were unable to identify such a microorganism able to trigger the onset of the disease and our results confirmed the presence of some aggressive bacterial strains characterizing the diseased specimens, in agreement with previous investigations. We cannot exclude that an amoeba might be the causative agent and primary cause of mortality [19].We cannot exclude that the causative agent could be dormant in the examined individuals, according to the Viable but Not-Culturable (VNC) hypothesis [46]. In particular, the VNC hypothesis proposes that some bacteria that do not form morphologically identifiable spores or cysts are, nevertheless, able to differentiate dormant cells and, in our case, some VNC strains could constitute a reservoir or even a direct source for the observed infection. In addition, the culture media employed here were not adequate for the isolation of cyanobacteria. Further investigations, based on the results of this study, should be focalized on the possible isolation of cyanobacteria from the green spots characterizing the first phases of the infection.

Our investigations also indicated (confirming the hypotheses of previous authors [47]), that these infections are a natural means to control the expansion of natural populations of sea urchins, which have a limited number of natural consumers. In fact, when the density of specimens in given areas increases, the probability of direct contacts also increases and this would facilitate direct contact among infected and healthy individuals, spreading the parasites [48]. Unfortunately, similar constraints may impact the production of intensive aquaculture plants, where higher densities of sea urchins and other echinoderms are cultured in narrow spaces.

Future investigations will be pointed to the identification of cyanobacteria possibly involved in the initiation of disease transmission, but the availability of a simple and fast treatment for diseased sea urchins, based on short baths, using plant-derived drugs, may represent an important step forward to control these diseases in aquaculture plants.

## Figures and Tables

**Table 1:** Bacterial strains isolated in the coelomic fluids or on the surface of the bodies of control sea urchins (in the absence of evident disease symptoms) and diseased sea urchins (in the presence of evident disease symptoms).

	Colony Number	Bacterial Strain	Identity (%)	Access Numbers (BLAST NCBI)
**Control sea urchins**				
Body surface	1	*Pseudoalteromonas* sp. 809C4	98.2	KP713471.1
	2	*Tenacibaculum* sp.	97.4	JX525366.1
Coelomic fluid	3	*Vibrio cortegadensis*	95.4	LS482976.1
	4	*Vibrio* sp.	97.7	LC416561.1
**Diseased sea urchins**				
Body surface	5	*Tenacibaculum* sp.	99.0	MK633875.1
	6	*Tenacibaculum soleae*	98.5	MN481031.1
	7	Uncultured *Vibrionaceae* bacterium	94.6	KF942331.1
	8	*Tenacibaculum* sp.	98.0	AM990737.1
	9	*Tenacibaculum* sp.	97.9	AM990757.1
	10	*Tenacibaculum* sp.	99.3	MN704063.1
	11	*Tenacibaculum* sp.	98.9	AM990737.1
	12	*Tenacibaculum* sp.	99.5	AM259865.1
Coelomic fluid	13	*Photobacterium* sp.	85.2	JX134425.1
	14 15	*Tenacibaculum* sp. *Tenacibaculum* sp.	98.6 99.2	AM990737.1 MN704063.1

## Data Availability

Not applicable.

## Supplementary Materials

The following supporting information can be downloaded at: https://www.mdpi.com/article/10.3390/microorganisms11030763/s1, Table S1: Total number of individuals collected in each month and number of diseased individuals found in each. Table S2: (A) Number of individuals dead in each of three replicates in treated and controls. (B) Survivorship rates calculated as a percentage of the total number of individuals treated and non-treated.

## References

[1] Guerinot M.L., Patriquin D.G. (1981). The association of N2-fixing bacteria with sea urchins. Mar. Biol..

[2] Holland N.D., Nealson K.H. (1978). The fine structure of the echinoderm cuticle and the subcuticular associated bacteria of echinoderms. Acta Zool..

[3] Kaneshiro E.S., Karp R.D. (1980). The ultrastructure of coelomocytes of the sea star *Dermasterias imbricata*. Biol. Bull..

[4] Johnson P.T. (1969). The coelomic elements of sea urchins (*Strongylocentrotus*) I. The normal coelomocytes; their morphology and dynamics in hanging drops. J. Invertebr. Pathol..

[5] Wardlaw A.C., Unkles S.E. (1978). Bactericidal activity of coelomic fluid from the sea urchin *Echinus esculentus*. J. Invertebr. Pathol..

[6] Messer L.I., Wardlaw A.C., Jangoux M. (1980). Separation of the Coelomocytes of *Echinus esculentus* by Density Gradient Centrifugation. Echinoderms Present and Past.

[7] Service M., Wardlaw A.C. (1984). Echinochrome-A as a bactericidal substance in the coelomic fluid of *Echinus esculentus* (L.). Comp. Biochem. Physiol. B.

[8] Jangoux M. (1987). Diseases of Echinodermata. I. Agents microorganisms and protistans. Dis. Aquat. Org..

[9] Maes P., Jangoux M. (1984). The bald-sea urchin disease: A biopathological approach. Helgol. Meeresunters.

[10] Maes P., Jangoux M., Fenaux L. (1986). La maladle de l’oursin-chauve: Ultrastructure des lesions et caracterisation de leur pigmentation. Ann. Inst. Oceanogr..

[11] Gilles K.W., Pearse J.S. (1986). Disease in sea urchins *Strongylocentrotus purpuratus*: Experimental infection and bacterial virulence. Dis. Aquat. Org..

[12] Giese A.C. (1961). Further studies on *Allocentrotus fragilis*, a deep-sea echinoid. Biol. Bull..

[13] Pearse J.S., Hines A.H. (1979). Expansion of a central California kelp forest following the mass mortality of sea urchins. Mar. Biol..

[14] Höbaus E., Fenaux L., Hignette M. (1981). Premières observations sur les lésions provoquées par une maladie affectant le test des oursins en Méditerranée occidentale. Rapp. P.-v. Réun. Commn Int. Explor. Scient. Meer. Méditerr..

[15] Nichols D. (1979). A nationwide survey of the British Sea Urchin *Echinus esculentus*. Progr. Underw. Sci..

[16] Scheibling R.E., Stephenson R.L. (1984). Mass mortality of *Strongylocentrotus droebachiensis* (Echinodermata: Echinoidea) off Nova Scotia, Canada. Mar. Biol..

[17] Shimizu M., Takaya Y., Ohsaki S., Kawamata K. (1995). Gross and histopathological signs of the spotting disease in the sea urchin *Strongylocentrotus intermedius*. Fish. Sci..

[18] Johnson P.T. (1970). Studies on diseased urchins from Point Loma. Kelp Habitat Improvement Project, Annual Report 1970–1971.

[19] Hernández J.C., Sangil C., Lorenzo-Morales J. (2020). Uncommon southwest swells trigger sea urchin disease outbreaks in Eastern Atlantic archipelagos. Ecol. Evol..

[20] Boudouresque C.F., Nedelec H., Sheperd S.A. (1980). The decline of a population of the sea urchin *Paracentrotus lividus* in the Bay of Port-Cros (Var, France). Sci. Rep. Port-Cros Natl. Park.

[21] Azzolina J.F. (1983). Evolution de la maladie de l’oursin comestible *Paracentrotus lividus* dans la baie de Port-Cros (Var, France). Rapp. PV Reun. Comm. Int. Explor. Sci. Mer Mediter. Monaco.

[22] Girard D., Clemente S., Toledo-Guedes K., Brito A., Hernández J.C. (2012). A mass mortality of subtropical intertidal populations of the sea urchin *Paracentrotus lividus*: Analysis of potential links with environmental conditions. Mar. Ecol..

[23] Feehan C.J., Scheibling R.E. (2014). Effects of sea urchin disease on coastal marine ecosystems. Mar. Biol..

[24] Salazar-Forero C.E., Reyes-Batlle M., González-Delgado S., Lorenzo-Morales J., Hernández J.C. (2022). Influence of Winter Storms on the Sea Urchin Pathogen Assemblages. Front. Mar. Sci..

[25] Clemente S., Lorenzo-Morales J., Mendoza J.C., López C., Sangil C., Alves F., Kaufmann M., Hernández J.C. (2014). Sea urchin *Diadema africanum* mass mortality in the subtropical eastern Atlantic: Role of waterborne bacteria in a warming ocean. Mar. Ecol. Progr. Ser..

[26] Miller R.J., Colodey A.G. (1983). Widespred mass mortalities of the green sea urchin in Nova Scotia, Canada. Mar. Biol..

[27] Mortensen T., Kolderup R.L. (1934). Sur une Algue Cyanophycée, Dactylococopsis Echini n. sp., Parasite Dans un Oursin.

[28] Baker-Austin C., Trinanes J.A., Salmenlinna S., Löfdahl M., Siitonen A., Taylor N.G., Martinez-Urtaza J. (2016). Heat wave–associated vibriosis, Sweden and Finland, 2014. Emerg. Infect. Dis..

[29] Grigioni S., Boucher-Rodoni R., Tonolla M., Peduzzi R. (1999). Symbiotic relations between bacteria and cephalopods. Boll. Soc. Ticin. Sci. Nat..

[30] Grigioni S., Boucher-Rodoni R., Demarta A., Tonolla M., Peduzzi R. (2000). Phylogenetic characterisation of bacterial symbionts in the accessory nidamental glands of the sepioid *Sepia officinalis* (Cephalopoda: Decapoda). Mar. Biol..

[31] Hamaguti M., Kawahara I., Usuki H. (1993). Mass mortality of *Pseudocentrotus depressus* caused by a bacterial infection in summer. Aquac. Sci..

[32] Zupo V., Fresi E. (1884). A study on the food web of the *Posidonia oceanica* ecosystem: Analysis of the gut contents of Echinoderms. International Workshop on Posidonia Oceanica Beds.

[33] Fagerli C.W., Stadniczeñko S.G., Pedersen M.F., Christie H., Fredriksen S., Norderhaug K.M. (2015). Population dynamics of *Strongylocentrotus droebachiensis* in kelp forests and barren grounds in Norway. Mar. Biol..

[34] Sadegh A.S., Sidoumou Z., Dia M., Pinchetti J.L., Bouaïcha N. (2002). Seasonal Occurrence of Cyanobacteria and First Detection of Microcystin-LR in Water Column of Foum-Gleita Reservoir, Mauritania. Environ. Process..

[35] Almanza V., Pedreros P., Laughinghouse H.D., Félez J., Parra O., Azócar M., Urrutia R. (2019). Association between trophic state, watershed use, and blooms of cyanobacteria in south-central Chile. Limnologica.

[36] Jones G.M., Scheibling R.E. (1985). *Paramoeba* sp. (Amoebida, Paramoebidae) as the possible causative agent of sea urchin mass mortality in Nova Scotia. J. Parasitol..

[37] Tajima K., Hirano T., Shimizu M., Ezura Y. (1997). Isolation and Pathogenicity of the Causative Bacterium of Spotting Disease of Sea Urchin *Strongylocentrotus intermedius*. Fish. Sci..

[38] Grech D., Mandas D., Farina S., Guala I., Brundu R., Cristo B., Panzalis P.A., Salati F., Carella F. (2022). *Vibrio splendidus* clade associated with a disease affecting *Paracentrotus lividus* (Lamarck, 1816) in Sardinia (Western Mediterranean). Invertebr. Pathol..

[39] Mortensen T., Rosevinge L.K. (1933). Sur une nouvelle algue. *Coccomyxa astericola*, parasite dans une asterie. Biol. Meddr..

[40] Jangoux M. (1984). Disease of echinoderm. Helgol. Meer..

[41] Baker-Austin C., Oliver J.D., Alam M., Ali A., Waldor M.K., Qadri F., Martinez-Urtaza J. (2018). *Vibrio* spp. infections. Nat. Rev. Dis. Prim..

[42] Oliver J.D. (2005). Wound infections caused by *Vibrio vulnificus* and other marine bacteria. Epidemiol. Infect..

[43] Vezzulli L., Colwell R.R., Pruzzo C. (2013). Ocean warming and spread of pathogenic vibrios in the aquatic environment. Microb. Ecol..

[44] Vevers H.G. (1951). The biology of *Asterias rubens* L. 11. Parasitization of the gonads by the ciliate *Orchitophyra stellarurn* Cepede. J. Mar. Biol. Assoc. UK.

[45] Delavault R., Leclerc M. (1969). Bactéries pathogènes découvertes chez *Asterina gibbosa* Penn. (échinoderme, astéride). CR Hebd. Séances Acad. Sci..

[46] Barer M.R., Gribbon L.T., Harwood C.R., Nwoguh C.E. (1993). The viable but non-culturable hypothesis and medical bacteriology. Rev. Med. Microbiol..

[47] Miller R.J. (1985). Sea urchin pathogen: A possible tool for biological control. Mar. Ecol. Prog. Ser..

[48] Scheibling R.E. (1984). Echinoids, epizootics and ecological stability in the rocky subtidal off Nova Scotia, Canada. Helgol. Meer. Res..

