# Supplementary material for: The “Bald Disease” of the Sea Urchin Paracentrotus lividus: Pathogenicity, Molecular Identification of the Causative Agent and Therapeutic Approach
Source: Microorganisms. 2023 Mar 16;11(3):763. doi: 10.3390/microorganisms11030763 (PMC10056887; doi:10.3390/microorganisms11030763)
Supplement: Supplementary file 1 [file microorganisms-11-00763-s001.zip › microorganisms-2251680-supplementary.pdf]

Month	Number of collected individuals	Number of diseased individuals
October	150	0
November	250	0
December	128	0
January	130	6
February	200	32
March	200	17
April	150	11
May	70	4
June	30	1
July	120	0
August	0	0
September	150	0

Table S1. Total number of individuals collected in each month and number of diseased individuals found in each collection

A)	Treated			Control		
**10 days**	0	0	0	1	3	3
**20 days**	0	0	0	3	4	4
**60 days**	0	0	1	5	5	5

Mortality (Number of individuals dead in each replicate)

B)	Treated			Control		
**10 days**	100	100	100	80	40	40
**20 days**	100	100	100	40	20	20
**60 days**	100	100	80	0	0	0

Survivorship %

Table S2: A) Number of individuals dead in each of three replicates in treated and controls. B) Survivorship rates calculated as a percentage on the total number of individuals treated and non treated
